# The Brazilian Portuguese version of the psoriasis epidemiology screening tool (PEST-bp) is reliable and accurate: a cross-sectional study from southern Brazil^؆^^؆؆^

**DOI:** 10.1016/j.abd.2025.501257

**Published:** 2025-12-26

**Authors:** Vanessa Thomé, Marcia Regina Rosa Scalcon, Denise Teresinha Antonelli da Veiga, Luciane Prado de Vargas, Patrícia Chagas, Camila Sales Fagundes, Gabriel Caruso Novaes Tudella, Mateus Diniz Marques, André Avelino Costa Beber, Raíssa Massaia Londero Chemello, Diego Chemello

**Affiliations:** a Postgraduate Program of Health Sciences, Universidade Federal de Santa Maria, Santa Maria, RS, Brazil; b Department of Clinical Medicine, Division of Dermatology, Universidade Federal de Santa Maria, Santa Maria, RS, Brazil; c Department of Clinical Medicine, Division of Rheumatology, Universidade Federal de Santa Maria, Santa Maria, RS, Brazil; d Department of Public Health, Universidade Federal de Santa Maria, RS, Brazil; e Graduation, Universidade Federal de Santa Maria, Santa Maria, RS, Brazil; f Department of Clinical Medicine, Division of Cardiology, Universidade Federal de Santa Maria, Santa Maria, RS, Brazil

**Keywords:** Arthritis, Psoriatic, Early diagnosis, Psoriasis

## Abstract

**Background:**

Psoriatic arthritis (PsA) remains diagnostically challenging in clinical practice. The Psoriasis Epidemiology Screening Tool - Brazilian Portuguese version (PEST-BP) offers a potential solution for simplified case identification.

**Objective:**

To evaluate the diagnostic accuracy of PEST-BP in detecting PsA among patients with psoriasis in a novel Southern Brazilian population.

**Methods:**

In this cross-sectional study, psoriasis patients from a dermatology clinic underwent dual assessment: PEST-BP screening and gold-standard rheumatologic evaluation using CASPAR criteria for PsA diagnosis. Statistical analyses included sensitivity, specificity, and ROC curve determination.

**Results:**

Among 100 patients, 21 (21%) met the CASPAR criteria for PsA. A PEST-BP score ≥ 3 showed the best diagnostic performance with 81% sensitivity, 79.7% specificity, and 80% overall accuracy (AUC = 0.845, p < 0.001). Patients with PsA had a significantly higher prevalence of dactylitis (38.1% vs. 11.4%; p = 0.004), nail psoriasis (66.7% vs. 35.4%; p = 0.01), and Psoriasis Area and Severity Index (PASI) ≥ 10 (42.9% vs. 19%; p = 0.023). In multivariate analysis, a PEST-BP score ≥ 3 (OR = 32.43; p < 0.001) and PASI ≥ 10 (OR = 9.26; p = 0.007) were independently associated with PsA.

**Study limitations:**

Single-center design in a tertiary care hospital and small sample size may overrepresent patients with severe disease.

**Conclusion:**

The PEST-BP is a reliable and accurate tool for PsA screening in Brazilian dermatology settings. Its simplicity and strong diagnostic performance support its integration into routine clinical practice for early PsA detection.

## Introduction

Psoriatic arthritis (PsA) is a chronic inflammatory disease that develops in some patients with psoriasis. Usually, there is a period of preclinical disease before the characteristic manifestations, which include arthritis, enthesitis, dactylitis, axial disease, or nail involvement.^1^, ^2^

Cutaneous lesions precede the development of musculoskeletal symptoms in the majority of patients. As a consequence, dermatologists and primary care physicians are in a favorable position to diagnose PsA.^3^ In fact, periodic screening is recommended by the most important medical societies in patients receiving topical treatment for psoriasis, those on systemic treatment, those with extensive affected areas, and those with nail or intergluteal involvement.

Because PsA can be difficult to diagnose, the CASPAR criteria were developed to help physicians identify people who have PsA. In the original study, these criteria presented both high sensitivity and specificity (0.987 and 0.914, respectively).^4^ However, there are several challenges and limitations when applying the criteria, particularly in cases of early or axial PsA.^5^

One simple way to increase the diagnosis of PsA is to use screening questionnaires, which are recommended by guidelines.^6^, ^7^, ^8^, ^9^ Several questionnaires for screening PsA have been developed over the last years. Some of these screening tests are complex and time-consuming, while others have different initial purposes rather than PsA screening.^1^ The PEST (Psoriasis Epidemiology Screening Tool) questionnaire was developed to screen for PsA in outpatients with psoriasis.^10^ This tool was originally published in English, and it was characterized by its quick applicability and easy understandability by patients. Recently, Mazzotti et al. translated the PEST into Portuguese (PEST-bp), validating it for the Brazilian population. They showed that the questionnaire is reliable in patients with psoriasis. According to their results, a cutoff score ≥ 3 has good sensitivity (84.6%) and specificity (63.3%) for the detection of PsA.^11^

Since there are no additional studies using the PEST-bp, the aim was to determine the accuracy of this tool in a distinct Brazilian population.

## Materials and methods

This was an observational cross-sectional study, consisting of 100 adult patients (≥18 years-old) with psoriasis. Patients were followed at the dermatology outpatient clinic at the Hospital Universitário de Santa Maria, Brazil. This is a university hospital in Santa Maria, Brazil, that is a reference for psoriasis care for a population of approximately 500,000 inhabitants. From March to September 2023, the authors evaluated all patients with psoriasis under follow-up at the dermatology outpatient clinic. The diagnosis of cutaneous psoriasis was made by two dermatologists certified by the Brazilian Society of Dermatology (LPV and RMLC), based on a clinical or histopathological examination of all patients. The exclusion criteria included individuals with a previous diagnosis of PsA or cognitive impairment that did not allow the questionnaire or the consent form to be completed. All patients were evaluated by a rheumatologist (MRRS) for the diagnosis of PsA using the CASPAR classification criteria, through detailed medical history and specialized physical examination, complemented, if necessary, by laboratory tests and imaging studies. The present study was approved by the local Research Ethics Committee.

To determine the required sample, the authors referred to the methodology employed by Mazzotti et al. In their cross-cultural validation and psychometric analysis, a sample size of 116 patients was estimated as necessary to achieve adequate statistical power. This calculation was based on an expected sensitivity of 97% and specificity of 79% for the PEST-bp questionnaire in detecting psoriatic arthritis (PsA), with a PsA prevalence of 20% among psoriasis patients. Cronbach’s alpha coefficient of 0.80 was considered for internal consistency, and a confidence interval of 95% was set. Utilizing these parameters, and accounting for a significance level (α) of 0.05 and a desired statistical power (1 - β) of 80%, the authors aim to recruit a minimum of 116 participants.

### Statistical analysis

The analyses were performed with the Statistical Package for Social Sciences (SPSS), version 21.0. The distribution of quantitative data was verified using the Kolmogorov-Smirnov test. The continuous variables were described as mean and standard deviation, or median and interquartile range, according to the distribution of data. Categorical variables were presented as absolute and relative values. For each PEST score, analysis of accuracy, sensitivity, and specificity was performed, and a Receiver Operating Characteristic (ROC) curve was constructed. The optimal cutoff point for the PEST score to identify patients at high risk for PsA was chosen using the Youden J index. A ROC curve > 0.7 was considered to indicate sufficient predictive accuracy. A multivariate logistic regression analysis was performed to determine the independent predictors associated with PsA.

**Fig. 1:**
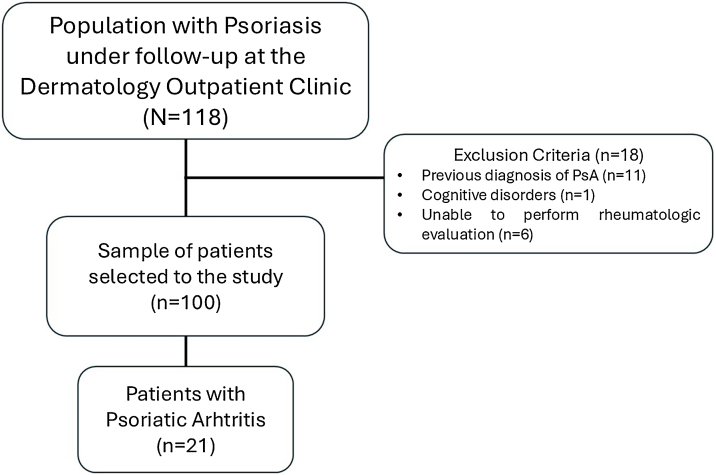
The flowchart of selection and initial results.

Flowchart of the study population (Fig. 1). Psoriasis patients were followed at the outpatient clinic (n = 118), with exclusions and the PsA identification process.

## Results

From the 118 patients with psoriasis under regular follow-up at the Dermatology Outpatient Clinics, eighteen were excluded from the present study for the following reasons: eleven had a priori diagnosis of PsA; one had cognitive disorders; and six patients were unable to undergo the complete rheumatological evaluation. The flow chart of selection and initial results is depicted in Fig. 1.

A total of 100 patients were eligible for the study. The mean age was 52.1 ± 14.8 years, 50 (50%) were female, and 18 (18%) were active smokers. The median Body Mass Index (BMI) was 29.8 kg/m^2^ (Interquartile Range (IR): 26.3–32.4 kg/m^2^). Seventy-five patients (75%) presented comorbidities, such as obesity in 39 (39%), and systemic arterial hypertension (SAH) in 37 (37%). A family history of psoriasis was present in 33 (33%). Eighty-five (85%) patients were users of Rheumatic Disease-Modifying Antirheumatic Drugs (DMARDs) at the inclusion in the study. Plaque psoriasis was the most common type with 73 (73%) patients, followed by palmoplantar in 17 (17%). The mean duration of the disease was 13.23 ± 9.41 years. Regarding psoriasis severity, 76 (76%) patients had mild disease with the median PASI score of 3.75 (IR: 1.13–9.3). A history of dactylitis was reported by 17 (17%) of the patients, and nail psoriasis was diagnosed in 42 (42%) cases. The median PEST score was 2 (IR: 1–3). Concomitant rheumatological diseases were present in 38 patients, with osteoarthritis occurring in 24 patients, followed by fibromyalgia in 5, gout in 3, and osteoporosis in 3. The baseline characteristics are shown in Table 1.

**Table 1:** Clinical and demographic characteristics of patients with psoriasis at the Dermatology Outpatient Clinic.

Characteristics	n (%)
Age (years)^a^	52.1 (14.8)
Female Sex	50 (50)
Obesity	39 (39)
SAH	37 (37)
Type 2 DM	18 (18)
Dyslipidemia	13 (13)
Depression	12 (12)
Cerebrovascular disease	2 (2)
Coronary artery disease	2 (2)
Body Mass Index (Kg/m^2^)^b^	28.3 (26.3 ‒ 32.4)
Active smoking	18 (18)
Former smoker	18 (18)
Family history of psoriasis	33 (33)
Using DMARDs	85 (85)
Psoriasis diagnosis (years)^a^	13.23 (9.41)
Psoriasis type	
Plaque	73 (73)
Palmoplantar	17 (17)
Guttate	6 (6)
Inverse	3 (3)
Pustulosis	1 (1)
Dactylitis	17 (17)
Nail psoriasis	42 (42)
PASI Score^b^	3.75 (1.13 – 9.3)
PASI Score < 10	76 (76)
PASI Score ≥ 10	24 (24)
PEST-bp Score^b^	2 (1 ‒ 3)
**Other rheumatologic diseases**	n = 38 (38)
Osteoarthritis	24 (63.2)
Fibromyalgia	5 (13.2)
Gout	3 (7.9)
Osteoporosis	3 (7.9)

SAH, systemic arterial hypertension; DM, diabetes mellitus; DMARDs, Disease-Modifying Rheumatologic Drugs.

a Values described as mean and Standard Deviation.

b Values described as median and interquartile range.

According to the CASPAR criteria, 21 (21%) of patients received the diagnosis of PsA. Their mean age was 55.7 ± 10.3 years, 12 (57.1%) were female, and the median BMI was 30 kg/m^2^ (IR: 26.6–32.6 kg/m^2^). The PASI score was ≥ 10 in 9 (42.9%) patients, corresponding to moderate to severe psoriasis. Dactylitis was present in 8 (38.1%), while nail psoriasis occurred in 14 (66.7%). Regarding joint involvement of PsA, the oligoarticular type was the most common, occurring in 10 (47.6%), followed by polyarticular in 7 (33.3%), and axial in 2 (9.5%). Among the 21 patients with PsA, the PEST-BP score was ≥ 3-points in 17 (81%). The main characteristics of patients with PsA are described in Table 2.

**Table 2:** Demographic, articular, and skin characteristics of patients diagnosed with psoriatic arthritis at the Dermatology Outpatient Clinic.

	n = 21 (%)
Age (years)^a^	55.7 (10.3)
Female sex	12 (57.1)
BMI (Kg/m^2^)^b^	30 (26.6 ‒ 32.6)
**Comorbidities**	
Obesity	10 (47.6)
HTN	7 (33.3)
DM Type 2	2 (9.5)
Dyslipidemia	5 (23.8)
Depression	3 (14.3)
Smoking	
Former smoker	1 (4.8)
Active smoker	5 (23.8)
Family history of psoriasis	7 (33.3)
Nail psoriasis	14 (66.7)
Dactylitis	8 (38.1)
DMARDs	18 (85.7)
PASI^b^	7.2 (0.8 ‒ 9.3)
**Severity of psoriasis**	
Up (PASI < 10)	12 (57.1)
Moderate/Severe (PASI ≥ 10)	9 (42.9)
**PEST-BP**	
< 3	4 (19)
≥ 3	17 (81)
**Joint pattern**	
Oligoarticular	10 (47.6)
Polyarticular	7 (33.3)
Oligoarticular/Axial	2 (9.5)
Axial	2 (9.5)

BMI, Body Mass Index; HTN, Hypertension; DM, Diabetes mellitus; DMARDs, Disease-Modifying Drugs; PASI, Psoriasis Area and Severity Index; PEST-BP, Psoriasis Epidemiology Screening Tool, Portuguese (Brazilian) version.

a Values described as mean and standard deviation.

b Values described as median and interquartile range.

In patients with PsA, there was a higher prevalence of dactylitis (38.1 vs. 11.4%; p = 0.004), and nail psoriasis (66.7% vs. 35.4%; p = 0.01). Also, there was a higher prevalence of PASI score ≥ 10 (42.9% vs. 19%; p = 0.023).

**Fig. 2:**
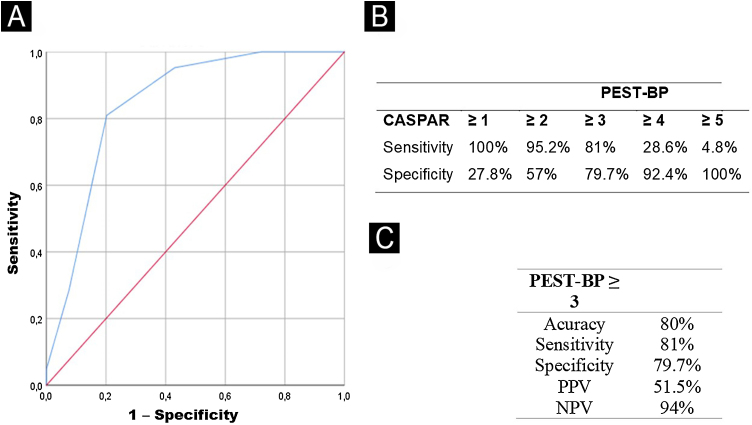
(A) The ROC curve shows sensitivity versus specificity of the scores used to discriminate patients with Psoriatic Arthritis (PsA). (B) Cumulative sensitivity and specificity for different cutoff points of the PEST-BP score according to CASPAR criteria. (C) Accuracy, sensitivity, specificity, Positive Predictive Value (PPV), and Negative Predictive Value (NPV) for the cutoff PEST-BP ≥3.

The ROC curve statistics showed the PEST-BP ≥ 3 presented the best cutoff for diagnosis of PsA, with the area under the curve of 0.845 (p < 0.001), accuracy of 80%, sensitivity of 81% and specificity of 79.7% (Fig. 2). The multivariate logistic regression revealed that a PEST-BP score ≥ 3 (OR = 32.43; p < 0.001) and a PASI score ≥ 10 (OR = 9.26; p = 0.007) were significantly associated with the diagnosis of PsA, as shown in Table 3.

**Table 3:** Multivariate logistic regression model for analysis of the association of variables with the diagnosis of psoriatic arthritis.

	Univariate Analysis		Multivariate Analysis	
	OR (95% IC)	p	OR (95% IC)	p
Age	1.06 (0.99‒1.13)	0.094		
Female sex	0.36 (0.06‒2.04)	0.247		
Comorbidities	0.54 (0.08‒3.63)	0.528		
Obesity	0.63 (0.14‒2.85)	0.550		
PASI ≥ 10	26.38 (2.85‒244.17)	0.004	9.26 (1.81‒47.35)	0.007
Ungual psoriasis	2.38 (0.42‒13.54)	0.327		
Dactylis	4.73 (0.91‒24.72)	0.065		
PEST ≥ 3	36.33 (4.43‒297.73)	0.001	32.43 (6.58‒159.92)	<0.001
DMRDs	1.05 (0.13‒8.36)	0.963		
Smoking	0.68 (0.13‒3.54)	0.649		
Family history of psoriasis	1.05 (0.20‒5.44)	0.958		

OR, Odds Ratio; 95% IC, 95% Confidence Interval; PASI, Psoriasis Area and Severity Score; PEST-BP, Psoriasis Epidemiology Screening Tool – Brazilian Portuguese; DMRDs, Disease-Modifying Rheumatic Drugs.

## Discussion

The PEST is a simple tool designed for screening PsA, enabling those at greatest risk to be referred to rheumatologic evaluation. In its original publication by Ibrahim et al., the cutoff determined by the ROC curve was ≥ 3, and the PEST presented a sensitivity of 92% and specificity of 78% for screening PsA.^10^ Recently, Mazzotti et al. translated and validated the PEST questionnaire into Portuguese, adapting it to the Brazilian population (PEST-bp). They observed an 81% accuracy with a cutoff ≥ 3 as suggestive of PsA, with a sensitivity of 84.6% and specificity of 63.3%.^11^ The present study, performed in a different Brazilian population, confirms the usefulness of the PEST-bp ≥3 for diagnosing PsA, with an accuracy of 80%, sensitivity of 81% and specificity of 79.7%.

There are other screening questionnaires developed for screening PsA, like the Toronto Psoriatic Arthritis Screen (ToPAS), the Psoriatic Arthritis Screening Evaluation (PASE), and EARP.^12^, ^13^, ^14^ The studied group chose to use the PEST tool due to its slightly superior performance, fast application, understandability by patients, and ease of application by physicians across different countries.^1^, ^15^, ^16^, ^17^

Regarding the severity of psoriasis, the authors observed that moderate to severe psoriasis (corresponding to a PASI score ≥ 10) is associated with a 9.26 times higher chance of PsA compared to mild disease. Similar results were observed by other authors, like Eder et al., who observed that severe psoriasis (PASI > 20) was a predictor of risk for PsA.^18^ A retrospective study by Wilson et al. showed that the risk of PsA was 2.24 times higher if skin was affected in more than 3 sites.^19^ Similarly, in a North American study with 27,220 patients who answered a questionnaire, Gelfand et al. observed that the prevalence of PsA was higher in patients who had more skin involvement.^20^ The association between psoriasis severity and PsA observed in several studies can be explained by a higher degree of systemic inflammation, which can be a trigger for musculoskeletal involvement.^8^

In the present study, the authors observed that there was a significantly higher prevalence of nail psoriasis in patients with PsA than in patients with cutaneous disease. These findings were also observed by other authors. For example, Wilson et al. observed that nail dystrophy was a predictor of risk for PsA.^19^ The association of nail psoriasis with PsA is thought to be due to the nail's proximity to the distal interphalangeal enthesis. When inflammation occurs, there is also a reflection in the nail apparatus.^21^

Dactylitis is also a pivotal manifestation of PsA, being part of the CASPAR criteria. It’s considered a marker of severe joint disease. In the present study, the authors observed a higher prevalence of dactylitis in patients with PsA and PEST-bp ≥ 3. Brockbanck et al. observed that radiological progression of psoriasis was more prominent in the group with dactylitis. Scriffignano et al. observed that patients with early-onset psoriasis were more likely to have dactylitis. Dactylitis is an early sign in 10% of patients with PsA, and half of patients with dactylitis have their first episode before the diagnosis of psoriatic joint disease.^22^, ^23^, ^24^

The authors acknowledge that the present study has limitations. First, this is a single-center study conducted in a tertiary hospital. As a consequence, the population had potentially more severe disease. Second, the sample size is considered small. Given the constraints of patient recruitment within the outpatient clinic, all eligible patients were initially considered for participation. However, following the application of inclusion and exclusion criteria, a total of 100 patients were successfully recruited for this study. While this sample size is smaller than the original study by Mazzotti et al. it represents the entirety of the accessible patient population within the study timeframe.^11^ Third, most patients were under systemic treatment with DMARDs at the time of the evaluation, which may have influenced the evaluation of the severity and extent of psoriasis and joint symptoms.

## Conclusion

Despite the limitations observed, the present work was able to confirm the high accuracy of the PEST-bp in tracking PsA. Considering the high rate of joint damage in the first years of the disease, the use of simple screening questionnaires becomes a tool for early detection of PsA, providing dermatologists with a unique opportunity to screen for this disease.

## ORCID ID

Vanessa Thomé: 0009-0001-9335-4777

Marcia Regina Rosa Scalcon: 0009-0005-0463-5833

Denise Teresinha Antonelli da Veiga: 0009-0000-8507-4073

Luciane Prado de Vargas: 0000-0001-8591-9603

Patrícia Chagas: 0000-0001-9808-2187

Camila Sales Fagundes: 0000-0002-6895-0702

Gabriel Caruso Novaes Tudella: 0009-0001-4140-6045

Mateus Diniz Marques: 0000-0002-8160-2569

André Avelino Costa Beber: 0000-0001-8952-6073

Raíssa Massaia Londero Chemello: 0000-0002-6824-0962

## Financial support

This work was supported by the Coordination for the Improvement of Higher Education Personnel – Brazil (CAPES) – Finance Code 001.

## Authors’ contributions

Vanessa Thomé: Conceptualization, data curation, formal analysis, investigation, project administration, resources.

Marcia Regina Rosa Scalcon: Conceptualization, investigation, validation, project administration, writing-original draft.

Denise Teresinha Antonelli da Veiga: Conceptualization, validation, visualization, project administration, writing-original draft.

Patrícia Chagas: Software, validation, writing-review & editing.

Camila Sales Fagundes: Resources, software, writing-review & editing.

Gabriel Caruso Novaes Tudella: Resources, writing-review & editing.

Mateus Diniz Marques: Validation, visualization, writing-review & editing.

Luciane Prado de Vargas: Investigation, methodology, visualization.

Raíssa Massaia Londero Chemello: Resources, visualization, writing-review & editing.

Diego Chemello: Conceptualization, funding acquisition, methodology, supervision, writing-original draft.

## Research data availability

The entire dataset supporting the results of this study was published in this article.

## Conflicts of interest

None declared.
